# Contributions of Upper- and Lower-Body Power to Countermovement Jump Performance with and Without Arm Swing

**DOI:** 10.3390/sports14070301

**Published:** 2026-07-15

**Authors:** Savanna Spires, Peri Rouillard, Andrew Hatchett, Iris Hatchett, Brock Renicks, Matthew Helms

**Affiliations:** 1 Department of Exercise and Sports Science, University of South Carolina Aiken, Aiken, SC 29801, USA; 2 Department of Anthropology, Tulane University, New Orleans, LA 70118, USA; 3 Department of Psychology, University of South Alabama, Mobile, AL 26688, USA

**Keywords:** countermovement jump, arm swing, peak power, anthropometrics, explosive performance

## Abstract

The countermovement jump (CMJ) is a common measure of lower-body power and neuromuscular performance. Incorporating an arm swing (AS) enhances CMJ outcomes, yet the contributions of anthropometrics, body composition (BC), and muscular power to performance are not fully understood in recreationally active adults. This study examined these factors in CMJ trials with and without AS. Thirty adults (15 males, 15 females; 18–25 years) performed CMJs on a force plate. Upper-body power was assessed via an 8 kg medicine ball throw, and lower-body power using a maximal cycling-based power test. Three-dimensional body scanning captured anthropometric data. Descriptive statistics, Pearson correlations, and hierarchical multiple regressions were conducted for the total sample and by sex. Jump height and peak power were significantly greater with AS than without (*p* < 0.001). Strong positive correlations were observed among jump- and power-related variables (r = 0.73–0.92). Body fat percentage showed moderate-to-strong negative associations with relative peak power, particularly in females. Regression analyses revealed that BC accounted for the largest proportion of variance in AS Peak Power, whereas upper-body power did not provide additional predictive value beyond anthropometrics and BC. These findings indicate that CMJ performance may be influenced primarily by lower-body mechanical capacity and BC, with adiposity consistently reducing relative power output. The results underscore the importance of BC in explosive performance training and support the use of AS-restricted jumps to isolate lower-body power during performance assessments.

## 1. Introduction

The CMJ is a widely used and well-validated assessment for evaluating lower-body power and neuromuscular performance across athletic, recreational, and clinical populations [1,2]. Consequently, it has become a cornerstone measure for athlete monitoring, performance evaluation, fatigue assessment, and training adaptation in both research and applied sport settings [3]. In particular, the CMJ is frequently used to assess readiness, fatigue, and training adaptations in power-based sports in which rapid force production is critical to success [4,5]. These sports include Olympic weightlifting, sprinting, jumping events, throwing disciplines, basketball, volleyball, American football, rugby, and soccer, all of which require athletes to generate high levels of force in minimal time during competitive actions.

The CMJ incorporates a rapid eccentric–concentric sequence that capitalizes on the stretch-shortening cycle (SSC), allowing it to reflect muscular strength, elastic energy storage, and intermuscular coordination [6,7]. This coupling of mechanical and neural factors closely resembles the demands of many sport-specific movements, such as sprint acceleration, change in direction, rebounding, and weightlifting pulls. Consequently, CMJ-derived variables such as jump height, peak power, and impulse are often used as proxies for an athlete’s ability to express explosive power during competition. Advances in force plate technology have further enhanced the value of the CMJ by enabling detailed examination of force–time characteristics, providing deeper insight into how force is produced, rather than simply how high an individual jumps.

In laboratory and clinical settings, the CMJ is often performed without an AS (hands akimbo) to isolate lower-limb contribution and improve test standardization. While this approach enhances internal validity, it may limit ecological relevance for sports in which coordinated whole-body movements dominate performance. In contrast, when an AS is permitted, CMJ performance is consistently enhanced, better reflecting the integrated nature of explosive athletic tasks [8,9]. Biomechanical analyses indicate that AS increases jump height by augmenting total system impulse, improving force transmission through the trunk, and increasing takeoff velocity [10,11]. These mechanisms align with performance strategies observed in power-based sports, where the upper and lower extremities act synergistically to maximize force output.

From an applied perspective, the magnitude of performance enhancement associated with AS is substantial. Improvements of approximately 10–30% in jump height have been reported when comparing AS to NAS conditions [9,12]. Such differences are meaningful in competitive sport contexts, where small changes in explosive performance can influence match outcomes, selection decisions, or training evaluations. However, these improvements are not uniform across individuals, suggesting that the effectiveness of AS may depend on a combination of anthropometric, neuromuscular, and coordinative factors that vary between individuals.

Anthropometric characteristics such as height and body mass have been linked to CMJ performance, particularly in relation to absolute and relative power production [13]. In power-based sports, where athletes often compete within weight classes or positional demands, body composition becomes a critical determinant of performance efficiency. Excess adiposity has been shown to negatively influence relative power output by increasing non-contributory mass that must be accelerated during explosive tasks [14,15]. These relationships highlight the importance of considering not only how much power an athlete can generate, but also how effectively that power is expressed relative to body mass.

Sex-based differences further complicate the interpretation of CMJ performance. Female participants often demonstrate stronger associations between body composition variables—particularly body fat percentage—and explosive performance outcomes such as jump height and relative peak power [14,15]. This relationship may be driven by differences in fat distribution and relative muscle mass, whereby a greater proportion of non-contractile tissue increases the mechanical load that must be accelerated during vertical jumping tasks, disproportionately affecting relative power output in females [10].

In addition to body composition, sex-related differences in muscle architecture and neuromuscular function may further influence CMJ performance. Females typically exhibit less absolute muscle cross-sectional area, reduced musculotendinous stiffness, and less maximal force production compared with males, which may alter force–time characteristics during the eccentric and concentric phases of the CMJ [7,16]. These structural and functional differences may necessitate alternative movement strategies, such as longer countermovement durations or reduced peak force expression, to achieve comparable jump heights. Consequently, performance outcomes in females may be more sensitive to changes in body composition and relative strength than in males, particularly when AS is restricted, and compensatory mechanisms are limited [16].

Neuromuscular activation strategies also appear to differ between sexes and may contribute to observed performance disparities. Previous research suggests that females may rely more heavily on proximal musculature and exhibit altered intermuscular coordination patterns during explosive tasks, potentially reflecting differences in motor unit recruitment, rate of force development, and coordination efficiency [17,18]. These factors may amplify the influence of anthropometric and body composition characteristics on CMJ performance, particularly in recreationally active populations where training histories and technical proficiency may vary widely.

From an applied perspective, recognizing sex-specific determinants of CMJ performance is critical for practitioners working in mixed-sex training environments. Assessment protocols that rely solely on absolute performance metrics may obscure meaningful differences in relative power expression and underlying mechanical limitations. Instead, incorporating relative power measures, AS-restricted jump testing, and body composition assessments may provide more informative insights for individualized program design. Targeted interventions emphasizing relative strength development, neuromuscular efficiency, and favorable body composition adaptations may be particularly effective for optimizing explosive performance in female athletes [18,19].

Despite substantial evidence supporting the performance benefits of arm swing (AS), relatively few studies have directly quantified upper-body power or examined its independent contribution to countermovement jump (CMJ) performance [20]. This represents a notable gap in the literature, especially given the importance of upper-body explosiveness in sports such as Olympic weightlifting, throwing events, and contact sports. It remains unclear whether upper-body power meaningfully predicts CMJ outcomes beyond anthropometric and lower-body factors, or whether the benefits of AS are primarily attributable to coordination, timing, and segmental sequencing rather than force-generating capacity alone [9]. Clarifying this distinction has important implications for both testing protocols and training emphasis in power-based athletes.

Therefore, the purpose of this study was to examine the contributing factors to CMJ power production under AS and NAS conditions in recreationally active adults. Specifically, this study aimed to evaluate the relationships among anthropometrics, body composition, upper-body power, and lower-body power, and to determine whether these variables differentially predict CMJ peak power. A secondary aim was to explore sex-specific patterns in these relationships, given known differences in body composition and neuromuscular characteristics. It was hypothesized that (1) AS would significantly enhance CMJ performance relative to NAS, (2) body composition would be a stronger predictor of relative peak power than upper-body power, and (3) body fat percentage would demonstrate stronger negative associations with jump performance in female participants.

## 2. Methods and Materials

### 2.1. Participants

Thirty recreationally active adults (15 males, 15 females) aged 18 to 25 years participated in this study. Participants were eligible if they engaged in at least two structured physical activity sessions per week and reported no upper- or lower-extremity injuries within the previous six months. Exclusion criteria included any history of neuromusculoskeletal disorders affecting movement or inability to safely perform maximal effort jumping or throwing tasks. All participants provided written informed consent prior to participation.

### 2.2. Testing Procedures

Anthropometric and body composition measurements were collected using a Fit3D ProScanner (Fit3D Inc., San Mateo, CA, USA). Participants wore form-fitting athletic clothing, removed footwear, and stood in a standardized position during scanning. Measures included height, body mass, lean mass, fat mass, and body fat percentage. Participants then completed a standardized dynamic warm-up consisting of light locomotor movements, dynamic mobility drills, and low-intensity plyometric exercises to prepare for maximal effort testing.

The countermovement jump (CMJ) assessment was performed using the VALD ForceDecks dual force plate system (VALD Performance, Brisbane, Australia). Ground reaction force data were sampled at 1000 Hz and processed using ForceDecks software (version 1.3.0 or the current laboratory version). Prior to testing, participants completed a standardized warm-up consisting of 5 min of light aerobic activity followed by dynamic lower-extremity movements and several submaximal practice jumps, consistent with recommended CMJ testing procedures [21].

Participants completed CMJs under two conditions: with arm swing (AS) and without arm swing (NAS). During the NAS condition, participants maintained an akimbo position with both hands placed on the hips throughout the movement to minimize upper-body contribution. For the AS condition, participants were instructed to use a natural arm swing to maximize jump performance. Three maximal-effort jumps were performed in each condition, with approximately 30–60 s of passive recovery provided between trials. Standardized verbal encouragement was given during each attempt, consistent with current recommendations for force plate-based jump assessments [21].

Vertical ground reaction force signals were low-pass filtered using a fourth-order zero-lag Butterworth filter (50 Hz cutoff frequency) prior to analysis. Body weight was determined from a quiet standing period preceding each jump. Movement onset was identified when vertical force deviated by more than 5 standard deviations from body weight, and take-off was detected when vertical force fell below 20 N, procedures that have been widely adopted in force plate analyses of CMJ performance [21,22]. Jump height was calculated using the impulse–momentum method, whereby take-off velocity was derived from the net vertical impulse and converted to jump height using the ballistic equation (*h* = *v*^2^/2*g*), the preferred approach because it is less susceptible to errors associated with flight-time calculations [4,21].

For each condition, the mean of the three trials was used for subsequent statistical analyses. Force plate-derived CMJ variables have demonstrated excellent measurement reliability, with intraclass correlation coefficients (ICC) generally exceeding 0.90 and coefficients of variation (CV) typically below 10% for jump height and other kinetic variables, supporting their use for evaluating lower-body neuromuscular performance [4,21].

Upper-body power was assessed using an 8 kg medicine ball incline throw. Participants were seated against a 45° incline bench with their head and upper back maintained in contact with the bench throughout the movement to minimize contributions from the trunk and lower extremities. From this standardized position, participants performed a maximal forward chest pass, and the horizontal distance traveled by the medicine ball was recorded. Three maximal trials were completed with 45–60 s of passive recovery between attempts, and the greatest throwing distance was retained for analysis. Medicine ball throw protocols performed from seated or inclined positions have demonstrated excellent reliability and validity as field-based assessments of upper-body explosive power across athletic and recreational populations [23,24]. Additionally, the seated medicine ball throw has been shown to provide reliable estimates of upper-body power while minimizing compensatory lower-body involvement [25].

Lower-body power was assessed using a Wattbike (Wattbike Ltd., Nottingham, UK) through a maximal cycling power protocol. Participants completed three 6 s maximal sprints against standardized resistance, with sufficient passive recovery between efforts to minimize fatigue. Peak power output (W) from the best trial performance was retained for statistical analysis. Short-duration maximal cycling tests performed on the Wattbike have been shown to provide valid and reliable measures of peak anaerobic power and demonstrate strong agreement with the laboratory-based Wingate Anaerobic Test while reducing the fatigue associated with longer testing protocols [26]. Participants completed the Wattbike lower-body power Test, consisting of three 6 s maximal-effort sprints performed on a Wattbike ergometer. Prior to testing, the bicycle was individually adjusted (e.g., saddle height, saddle fore-aft position, and handlebar height) according to standardized fitting procedures to ensure proper biomechanics and participant comfort. Participants then completed a standardized 5 min warm-up at a self-selected, light-to-moderate intensity. Following the warm-up, participants performed three 6 s maximal sprints, with 1 min of passive recovery between each bout. Strong verbal encouragement was provided during each effort to promote maximal performance. Upon completion of the final sprint, participants performed a 5 min cool-down at a light, self-selected intensity.

### 2.3. Data Analysis

Descriptive statistics were calculated for all variables for the total sample and stratified by sex. Pearson product–moment correlations were used to examine relationships among anthropometric, body composition, and performance variables. Hierarchical multiple regression analyses were conducted to identify predictors of AS Peak Power normalized to body mass, with height entered first, followed by body composition variables, and then performance variables. Pearson product–moment correlation coefficients (*r*) were calculated to examine the relationships among anthropometric, body composition, and performance variables. The magnitude of correlation coefficients was interpreted according to the recommendations of [27]: trivial (|*r*| < 0.10), small (|*r*| = 0.10–0.29), moderate (|*r*| = 0.30–0.49), large (|*r*| = 0.50–0.69), very large (|*r*| = 0.70–0.89), nearly perfect (|*r*| = 0.90–0.99), and perfect (*r* = 1.00). Both the strength and direction of statistically significant relationships were interpreted, and coefficients of determination (*r*^2^) were reported for selected key relationships to facilitate interpretation of the proportion of shared variance between variables. Statistical significance was set at *p* < 0.05.

## 3. Results

### 3.1. Descriptive Statistics

Descriptive characteristics for the total sample (N = 30) and by sex are presented in Table 1. The sample consisted of 15 male and 15 female participants. Overall, participants demonstrated a mean lower-body power output of 2062.2 ± 611.5 W and a medicine ball throw distance of 98.9 ± 23.1 in. Mean height, body mass, body fat percentage, lean mass, and fat mass for the total sample were 67.9 ± 3.9 in, 159.7 ± 43.7 lb, 22.4 ± 8.0%, 122.1 ± 26.1 lb, and 37.6 ± 23.0 lb, respectively.

Male participants were taller (70.3 ± 2.3 vs. 65.5 ± 3.6 in), heavier (178.8 ± 47.6 vs. 140.6 ± 30.0 lb), and possessed greater lean mass (139.1 ± 24.0 vs. 105.0 ± 14.5 lb) than female participants, whereas females exhibited a higher mean body fat percentage (24.2 ± 6.9% vs. 20.5 ± 8.7%). Mean fat mass was similar between groups (39.7 ± 28.0 vs. 35.6 ± 17.3 lb).

Performance measures also differed by sex. Male participants produced greater lower-body power (2232.1 ± 628.3 vs. 1892.2 ± 563.7 W) and longer medicine ball throw distances (115.9 ± 18.8 vs. 81.9 ± 11.4 in) than female participants. During the countermovement jump performed without arm swing (NAS), males demonstrated greater jump height (35.9 ± 11.5 vs. 25.5 ± 5.8), peak power (62.63 ± 9.4 vs. 42.48 ± 4.8), and peak power relative to body mass (52.2 ± 10.3 vs. 41.3 ± 5.7). Similar trends were observed for the countermovement jump performed with arm swing (AS), with males demonstrating higher jump height (49.0 ± 25.3 vs. 30.4 ± 6.9), peak power (70.56 ± 9.47 vs. 54.29 ± 3.8), and peak power relative to body mass (64.4 ± 16.7 vs. 47.9 ± 7.4). Across the total sample, AS countermovement jumps produced greater jump heights and peak power values than NAS jumps.

### 3.2. Correlational Analyses

Pearson product–moment correlations were examined among anthropometric, body composition, and performance variables for the total sample and stratified by sex (Table 2). For the total sample, very large-to-nearly perfect positive associations were observed among all jump- and power-related variables (*r* = 0.73–0.92, *p* < 0.001), corresponding to approximately 53–85% shared variance. Notably, no-arm-swing jump height was strongly associated with no-arm-swing peak power (*r* = 0.91, *r*^2^ = 0.83), while arm-swing jump height demonstrated a similarly strong relationship with relative peak power (*r* = 0.89, *r*^2^ = 0.79). Body fat percentage exhibited large negative associations with relative peak power measures, including no-arm-swing peak power relative to body mass (*r* = −0.62, *r*^2^ = 0.38) and arm-swing peak power relative to body mass (*r* = −0.55, *r*^2^ = 0.30). Lean mass was nearly perfectly associated with medicine ball performance (*r* = 0.90, *r*^2^ = 0.81), indicating that greater lean mass was strongly related to upper-body power output. Height was also positively associated with lean mass and medicine ball performance.

Sex-specific analyses revealed distinct patterns in the relationships among body composition and performance variables. Among males, jump and power variables remained strongly interrelated, with correlations ranging from large to nearly perfect (e.g., no-arm-swing jump height and no-arm-swing peak power, *r* = 0.86; arm-swing jump height and relative peak power, *r* = 0.85). However, body composition variables demonstrated fewer significant relationships with performance outcomes. Although body fat percentage was negatively associated with no-arm-swing peak power relative to body mass (*r* = −0.58, *p* < 0.05), other associations between adiposity and performance were generally weaker and did not consistently reach statistical significance.

In contrast, females demonstrated stronger and more consistent associations between body composition and performance. Body fat percentage exhibited large negative relationships with relative peak power measures, including no-arm-swing peak power relative to body mass (*r* = −0.69, *r*^2^ = 0.48) and arm-swing peak power relative to body mass (*r* = −0.67, *r*^2^ = 0.45), indicating that greater adiposity was associated with lower relative power production. Additionally, jump height and peak power variables were nearly perfectly related (e.g., no-arm-swing jump height and no-arm-swing peak power, *r* = 0.95, *r*^2^ = 0.90; arm-swing jump height and relative peak power, *r* = 0.95, *r*^2^ = 0.90). Collectively, these findings suggest that body composition may play a more influential role in lower-body performance among females than males within the present sample.

#### Paired-Sample *t*-Test Analysis

As displayed in Table 3, apaired-sample *t*-test was conducted to compare countermovement jump height with arm swing (AS jump height) and without arm swing (NAS jump height). Results indicated a statistically significant difference between conditions, t(29) = 4.10, *p* < 0.001. Jump performance was higher in the arm-swing condition (M = 39.73, SD = 20.54) compared with the no-arm-swing condition (M = 30.67, SD = 10.38), yielding a mean difference of 9.06 units (95% CI [4.54, 13.59]).

The association between conditions was strong and positive (r = 0.898, *p* < 0.001), indicating that individuals who performed well in one condition tended to perform well in the other. The magnitude of the difference was moderate-to-large, with a standardized effect size of Cohen’s d = 0.75 (Hedges’ g = 0.73), suggesting a practically meaningful performance enhancement associated with arm swing during the countermovement jump.

**Table 3:** Results of a paired-sample *t*-test analysis comparing CMJ with and without arm swing.

Variable Pair	M	SD	r	Mean Difference	*t*	df	*p*	95% CI
AS vs. NAS CMJ	39.73	20.54	0.898	9.06	4.1	29	<0.001	[4.54, 13.59]
	30.67	10.38						

### 3.3. Regression Analyses

Hierarchical multiple regression analyses were conducted with AS Peak Power as the dependent variable. Predictors were entered in three stages: (1) height, (2) body composition variables (fat mass, lean mass, body fat percentage), and (3) performance variables (lower-body power, medicine ball performance).

For the total sample, height emerged as a significant baseline predictor, explaining 16.9% of the variance. The addition of body composition variables significantly improved the model, increasing explained variance to 45.2%. The final addition of performance variables did not significantly enhance model fit, yielding a final R^2^ of 0.481. Notable multicollinearity was observed among body composition variables.

Sex-specific regression analyses demonstrated divergent patterns. In one subgroup, no model reached statistical significance, whereas in the other subgroup, body composition accounted for a substantial proportion of explained variance (>50%), and height emerged as a significant suppressor variable in the final model. Across all models, performance variables did not contribute significant unique variance beyond anthropometric and body composition measures.

#### Regression Analysis

A hierarchical regression analysis was conducted to examine whether medicine ball performance (MBavg), bike power peak average (BPPAvg), and body fat percentage (BFPer) predicted arm-swing countermovement jump height (AS jump height) can be seen in Table 4. The overall regression model was not statistically significant, *F*(3, 26) = 1.93, *p* = 0.150, accounting for 18.2% of the variance in AS jump height (*R*^2^ = 0.182, adjusted *R*^2^ = 0.088).

Although the omnibus model did not reach statistical significance, inspection of individual predictors indicated differential associations with AS jump height. Medicine ball performance was not a significant predictor (*B* = 0.168, SE = 0.167, β = 0.189, *t* = 1.005, *p* = 0.324). Bike power peak average demonstrated a positive association with AS jump height and reached statistical significance (*B* = 0.022, SE = 0.018, β = 0.262, *t* = 1.214, *p* = 0.035). In contrast, body fat percentage was negatively associated with AS jump height and was statistically significant (*B* = −0.350, SE = 0.538, β = −0.136, *t* = −0.651, *p* = 0.021). The intercept was also statistically significant (*B* = 12.759, SE = 24.441, *t* = 0.522, *p* = 0.045).

**Table 4:** Summary of hierarchical regression models predicting AS Peak Power.

Source	SS	df	MS	F	*p*	
Regression	2227.246	3	742.415	1.928	0.15	
Residual		10,013.481	26	385.134		
Total		12,240.727	29			
Predictor		B	SE	β	t	*p*
Constant		12.759	24.441	—	0.522	0.045
MBavg		0.168	0.167	0.189	1.005	0.324
BPPAvg		0.022	0.018	0.262	1.214	0.035
BFPer		−0.350	0.538	−0.136	−0.651	0.021

AS jump height = arm-swing countermovement jump height; MBavg = medicine ball average throw distance; BPPAvg = body power peak average; BFPer = body fat percentage. *B* = unstandardized regression coefficient; SE = standard error; β = standardized coefficient. Statistical significance was set at *p* < 0.05.

## 4. Discussion

Despite substantial evidence demonstrating that an arm swing (AS) enhances countermovement jump (CMJ) performance, relatively few studies have examined the independent contribution of upper-body power after accounting for anthropometric characteristics, body composition, and lower-body power. Consequently, it remains unclear whether improvements in CMJ performance with AS are primarily attributable to upper-body force-generating capacity or to enhanced movement coordination and segmental sequencing. Addressing this gap is important because CMJ testing is widely used by coaches, clinicians, and sport scientists to evaluate neuromuscular performance, monitor training adaptations, and inform rehabilitation and performance programming. A better understanding of the factors that influence CMJ performance under AS and no arm swing (NAS) conditions may improve the interpretation of jump assessments and guide more targeted training interventions.

The purpose of this study was to examine the contributing factors to jump power production, with particular emphasis on the role of anthropometrics, body composition, and upper- and lower-body power measures in jumps performed with and without an AS. The primary findings indicate that jump power is strongly influenced by lower-body mechanical capacity across conditions, while body composition—particularly adiposity—emerges as a key moderating factor, especially when the AS is restricted. Collectively, these results suggest that while the AS enhances jump performance through coordination and force transfer, underlying physical characteristics remain central determinants of power output.

Consistent with prior literature, very strong correlations were observed among jump height, impulse momentum, and peak power measures across AS conditions. These findings align with biomechanical models demonstrating that AS augments vertical jump performance by increasing system impulse, enhancing center-of-mass displacement, and allowing greater force production at the hip and knee extensors [8,10]. The high shared variance between AS and NAS jump metrics in the present study suggests that AS primarily may amplify existing lower-body power rather than fundamentally alter the performance hierarchy among individuals.

Body composition emerged as a meaningful contributor to jump power production, particularly for relative (body–mass-normalized) outcomes. Across the total sample, body fat percentage demonstrated moderate to strong negative relationships with both AS and NAS peak power. These findings support previous work showing that excess non-functional mass imposes mechanical constraints on vertical displacement and limits relative power output [14,15]. Notably, these negative associations were more pronounced in AS-restricted conditions, suggesting that when compensatory strategies such as arm contribution are removed, the detrimental influence of adiposity on jump performance becomes more apparent.

Sex-stratified analyses revealed distinct patterns in the contribution of body composition to jump power. Female participants demonstrated stronger and more consistent negative associations between body fat percentage and all jump power variables, regardless of AS condition. This finding aligns with previous research indicating that relative strength and body composition exert a greater influence on explosive performance in female athletes [16,17]. In contrast, male participants exhibited weaker and less consistent relationships between body composition and jump performance, suggesting that greater absolute strength and muscle mass may partially offset the mechanical disadvantages associated with higher fat mass during jumping tasks.

Hierarchical regression analyses further clarified the relative importance of contributing factors. While height served as a modest baseline predictor of jump power, the addition of body composition variables accounted for the largest increase in explained variance, whereas upper-body performance measures (medicine ball throw and lower-body power) failed to provide meaningful unique contributions. These findings suggest that although the AS relies on coordinated upper-body motion, upper-body power alone may not independently predict jump power once lower-body and anthropometric factors are considered. This supports previous biomechanical evidence that the AS benefit is largely derived from timing, coordination, and force transmission rather than upper-body strength per se [9].

From an applied sport science perspective, the interpretation of these findings aligns strongly with established literature in neuromuscular performance, plyometrics, and jump biomechanics. Collectively, the results highlight that vertical jump performance is not solely a function of task execution (e.g., arm use) but rather emerges from the interaction of lower-body force production capacity, intermuscular coordination, and morphological characteristics such as body composition [27,28,29,30,31].

A primary consideration is the role of lower-body force production as a key driver of vertical jump performance improvements. Extensive evidence indicates that enhancements in maximal and rapid force production—particularly in the lower extremities—are strongly associated with improvements in countermovement jump (CMJ) performance [18,19]. Training interventions that improve rate of force development (RFD), neuromuscular efficiency, and stretch-shortening cycle utilization consistently translate into increased jump height and power output [31,32]. In this context, both AS (arm-swing) and AS-restricted conditions ultimately reflect the underlying capacity of the neuromuscular system to generate force against the ground. Thus, improvements observed in either condition are likely underpinned by adaptations in muscular strength, tendon stiffness, and motor unit recruitment strategies rather than task-specific mechanics alone.

Body composition further contributes meaningfully to jump performance outcomes. A lower relative fat mass and higher lean mass are consistently associated with superior jump height and power, primarily due to improved force-to-mass ratio [18,19]. Because vertical jumping is a body–weight-dependent task, even modest improvements in relative strength (i.e., force production relative to body mass) can significantly enhance performance. This helps explain why changes in jump performance often co-occur with strength gains and favorable shifts in body composition, particularly in training populations engaged in resistance and plyometric training interventions [32].

The distinction between AS-restricted and AS conditions is particularly important from an assessment standpoint. Restricting arm swing reduces the contribution of upper-body momentum and minimizes compensatory movement strategies, thereby isolating lower-extremity force production capacity more directly. Research has shown that arm swing can increase vertical jump height by 10–20% through enhanced impulse generation and improved coordination of segmental momentum transfer [8,9]. Consequently, AS-restricted jumps (e.g., hands-on-hips CMJ) may provide a more “pure” measure of lower-body power by limiting elastic and coordinative contributions from the upper body and trunk. This makes them particularly useful for identifying mechanical limitations such as force production deficits, impaired eccentric–concentric transition efficiency, or suboptimal impulse characteristics.

In contrast, AS jumps may better reflect sport-specific performance demands, where whole-body coordination and segmental interaction are essential for maximizing output. Many athletic tasks—such as rebounding, spiking, or sprint-based jump actions—naturally incorporate arm swing and full-body integration to optimize impulse. The coordinated contribution of the upper extremities increases total system momentum and enhances take-off velocity through improved mechanical energy transfer [9,32]. From this perspective, AS jumps may provide higher ecological validity for sport performance assessment, even if they introduce additional variability due to coordination skill.

Collectively, these findings add to the current body of literature by reinforcing that optimal jump performance is influenced by the interaction of multiple neuromuscular, biomechanical, and morphological factors, supporting the use of multifactorial training approaches. Strength development remains foundational, particularly for improving maximal force output and RFD characteristics [19]. However, technical coordination—including effective arm swing timing and trunk control—plays a critical role in translating force into usable impulse during dynamic sport tasks [1]. Therefore, optimal programming should integrate heavy resistance training, plyometrics, and sport-specific coordination drills to maximize transfer to performance. Additionally, monitoring both AS and AS-restricted jump outcomes can provide practitioners with complementary insights: one reflecting maximal lower-body capacity and the other reflecting integrated athletic performance.

## 5. Conclusions

This study’s results demonstrated that lower-body mechanical capacity and body composition may be primary determinants of jump power, with adiposity exerting a particularly strong influence on relative performance when the arm swing is restricted. While the arm swing enhances jump outcomes through coordination and force transfer, it largely appeared to amplify existing lower-body power rather than altering the performance hierarchy among individuals. Sex-specific analyses highlight that body composition may play a more pronounced role in female participants, emphasizing the importance of relative strength and lean mass in explosive movements. Medicine ball throw performance did not explain additional variance in CMJ performance beyond anthropometric and body composition variables.

These findings suggest that targeted interventions aimed at improving lower-body strength, optimizing body composition, and refining technical coordination are essential for maximizing jump power, and that arm-swing-restricted testing can effectively isolate true lower-body capacity.

## Figures and Tables

**Table 1:** Descriptive characteristics of the total sample and stratified by sex.

Variable	Total Sample (N = 30)	Male (n = 15)	Female (n = 15)
Lower-Body Power (W)	2062.2 ± 611.5	2232.1 ± 628.3	1892.2 ± 563.7
Medicine Ball Average (in)	98.9 ± 23.1	115.9 ± 18.8	81.9 ± 11.4
Height (in)	67.9 ± 3.9	70.3 ± 2.3	65.5 ± 3.6
Body Mass (BM) (lb)	159.7 ± 43.7	178.8 ± 47.6	140.6 ± 30.0
Body Fat (%)	22.4 ± 8.0	20.5 ± 8.7	24.2 ± 6.9
Lean Mass (lb)	122.1 ± 26.1	139.1 ± 24.0	105.0 ± 14.5
Fat Mass (lb)	37.6 ± 23.0	39.7 ± 28.0	35.6 ± 17.3
NAS Jump Height	30.7 ± 10.4	35.9 ± 11.5	25.5 ± 5.8
NAS Peak Power	52.3 ± 6.7	62.63 ± 9.4	42.48 ± 4.8
NAS Peak Power/BM	46.7 ± 9.9	52.2 ± 10.3	41.3 ± 5.7
AS Jump Height	39.7 ± 20.5	49.0 ± 25.3	30.4 ± 6.9
AS Peak Power	61.91 ± 18.78	70.56 ± 9.47	54.29 ± 3.8
AS Peak Power/BM	56.2 ± 15.2	64.4 ± 16.7	47.9 ± 7.4
Variable	Total Sample (N = 30)	Male (n = 15)	Female (n = 15)
Lower-Body Power (W)	2062.2 ± 611.5	2232.1 ± 628.3	1892.2 ± 563.7
Medicine Ball Average (in)	98.9 ± 23.1	115.9 ± 18.8	81.9 ± 11.4
Height (in)	67.9 ± 3.9	70.3 ± 2.3	65.5 ± 3.6
Body Mass (BM) (lb)	159.7 ± 43.7	178.8 ± 47.6	140.6 ± 30.0
Body Fat (%)	22.4 ± 8.0	20.5 ± 8.7	24.2 ± 6.9
Lean Mass (lb)	122.1 ± 26.1	139.1 ± 24.0	105.0 ± 14.5
Fat Mass (lb)	37.6 ± 23.0	39.7 ± 28.0	35.6 ± 17.3
NAS Jump Height	30.7 ± 10.4	35.9 ± 11.5	25.5 ± 5.8
NAS Peak Power	52.3 ± 6.7	62.63 ± 9.4	42.48 ± 4.8
NAS Peak Power/BM	46.7 ± 9.9	52.2 ± 10.3	41.3 ± 5.7
AS Jump Height	39.7 ± 20.5	49.0 ± 25.3	30.4 ± 6.9
AS Peak Power	61.91 ± 18.78	70.56 ± 9.47	54.29 ± 3.8
AS Peak Power/BM	56.2 ± 15.2	64.4 ± 16.7	47.9 ± 7.4

Note: Mean ± SD, BM = body mass, NAS = no arm swing, AS = arm swing.

**Table 2:** Selected significant correlations (|r| ≥ 0.50) by group.

Variable Pair	Total Sample	Male	Female
NAS Jump Height–NAS Peak Power	0.91 **	0.86 **	0.95 **
AS Jump Height–AS Peak Power/BM	0.89 **	0.85 **	0.95 **
Body Fat %–NAS Peak Power/BM	−0.62 **	−0.58 *	−0.69 **
Body Fat %–AS Peak Power/BM	−0.55 **	ns	−0.67 **
Lean Mass–Medicine Ball	0.90 **	0.86 **	0.70 **

Note: ** *p* < 0.01; * *p* < 0.05; NAS = no arm swing, AS = arm swing, BM = body mass.

## Data Availability

The data presented in this study are available on request from the corresponding author due to ethical restriction.

## References

[1] Markovic G., Dizdar D., Jukic I., Cardinale M. (2004). Reliability and factorial validity of squat and countermovement jump tests. J. Strength Cond. Res..

[2] Petrigna L., Karsten B., Marcolin G., Paoli A., D’Antona G., Palma A., Bianco A. (2019). A review of countermovement jump performance assessment in sport science. J. Hum. Kinet..

[3] Pérez-Castilla A., Weakley J., García-Pinillos F., Rojas F.J., García-Ramos A. (2021). Influence of countermovement depth on the countermovement jump-derived reactive strength index modified. Eur. J. Sport Sci..

[4] Claudino J.G., Cronin J., Mezêncio B., McMaster D.T., McGuigan M., Tricoli V., Amadio A.C., Serrão J.C. (2017). The countermovement jump to monitor neuromuscular status: A meta-analysis. J. Sci. Med. Sport.

[5] Gathercole R., Sporer B., Stellingwerff T., Sleivert G. (2015). Effect of acute fatigue and training adaptation on countermovement jump performance in elite snowboard cross athletes. J. Strength Cond. Res..

[6] Asmussen E., Bonde-Petersen F. (1974). Storage of elastic energy in skeletal muscles in man. Acta Physiol. Scand..

[7] Komi P.V. (2000). Stretch-shortening cycle: A powerful model to study normal and fatigued muscle. J. Biomech..

[8] Harman E.A., Rosenstein M.T., Frykman P.N., Rosenstein R.M. (1990). The effects of arms and countermovement on vertical jumping. Med. Sci. Sports Exerc..

[9] Lees A., Vanrenterghem J., De Clercq D. (2004). Understanding how an arm swing enhances performance in the vertical jump. J. Biomech..

[10] Bobbert M.F., Gerritsen K.G.M., Litjens M.C.A., Van Soest A.J. (1996). Why is countermovement jump height greater than squat jump height?. Med. Sci. Sports Exerc..

[11] Cheng K.B., Wang C.H., Chen H.C., Wu C.D., Chiu H.T. (2008). The mechanisms that enable arm motion to enhance vertical jump performance—A simulation study. J. Biomech..

[12] Caruso J.F., Daily J.S., McLagan J.R., Shepherd C.M., Olson N.M., Marshall M.R., Taylor S.T. (2012). Anthropometry as a predictor of vertical jump performance. J. Strength Cond. Res..

[13] Jaric S., Mirkov D., Markovic G. (2005). Normalizing physical performance tests for body size: A proposal for standardization. J. Strength Cond. Res..

[14] Markovic G., Jaric S. (2007). Is vertical jump height a body size–independent measure of muscle power?. J. Sports Sci..

[15] Ferreira L.C., Barbosa T.M., Neiva H.P. (2020). Effect of arm swing on vertical jump performance in males and females. J. Sports Sci..

[16] Thomas C., Comfort P., Chiang C.Y., Jones P.A. (2015). Relationship between isometric mid-thigh pull variables and sprint and change of direction performance in collegiate athletes. J. Strength Cond. Res..

[17] Suchomel T.J., Nimphius S., Stone M.H. (2016). The importance of muscular strength in athletic performance. Sports Med..

[18] McGuigan M.R., Cormack S.J., Gill N.D. (2012). Strength and power profiling of athletes: Selecting tests and how to use the information for program design. Strength Cond. J..

[19] Haff G.G., Nimphius S. (2012). Training principles for power. Strength Cond. J..

[20] Mosier E.M., Fry A.C., Lane M.T. (2019). Kinetic contributions of the upper limbs during countermovement vertical jumps with and without arm swing. J. Strength Cond. Res..

[21] Clemons J.M., Campbell B., Jeansonne C. (2010). Validity and reliability of a new test of upper body power. J. Strength Cond. Res..

[22] Herbert P., Sculthorpe N., Baker J.S., Grace F.M. (2015). Validation of a six second cycle test for the determination of peak power output. Res. Sports Med..

[23] Sayers M.G.L., Bishop S. (2017). Reliability of a new medicine ball throw power test. J. Appl. Biomech..

[24] Strand K.L., Ly A.S., Barry S.S., Liscano J.A., Trebotich T.L., Martin-Diala C., Martin E., Signorile J.F. (2023). Validity and reliability of the seated medicine ball throw as a measure of upper body power in older women. J. Strength Cond. Res..

[25] Cormie P., McGuigan M.R., Newton R.U. (2011). Developing maximal neuromuscular power: Part 1—Biological basis of maximal power production. Sports Med..

[26] Hara M., Shibayama A., Takeshita D., Hay D.C., Fukashiro S. (2006). The effect of arm swing on lower extremities in vertical jumping. J. Biomech..

[27] Hopkins W.G., Marshall S.W., Batterham A.M., Hanin J. (2009). Progressive statistics for studies in sports medicine and exercise science. Med. Sci. Sports Exerc..

[28] Nishiumi D., Nishioka T., Saito H., Kurokawa T., Hirose N. (2023). Associations of eccentric force variables during jumping and eccentric lower-limb strength with vertical jump performance: A systematic review. PLoS ONE.

[29] Suchomel T.J., Nimphius S., Bellon C.R., Stone M.H. (2018). The importance of muscular strength: Training considerations. Sports Med..

[30] Carlock J.M., Smith S.L., Hartman M.J., Morris R.T., Ciroslan D.A., Pierce K.C., Newton R.U., Harman E.A., Sands W.A., Stone M.H. (2004). The relationship between vertical jump power estimates and weightlifting ability: A field test approach. J. Strength Cond. Res..

[31] Nikolaidis P.T., Afonso J., Busko K. (2016). Relationship between body composition and athletic performance. Asian J. Sports Med..

[32] van Hooren B., Zolotarjova J. (2017). The difference between countermovement and squat jump performance: A review of underlying mechanisms. J. Strength Cond. Res..

